# Fungal α-1,3-Glucan as a New Pathogen-Associated Molecular Pattern in the Insect Model Host *Galleria mellonella*

**DOI:** 10.3390/molecules26165097

**Published:** 2021-08-23

**Authors:** Sylwia Stączek, Agnieszka Zdybicka-Barabas, Iwona Wojda, Adrian Wiater, Paweł Mak, Piotr Suder, Krzysztof Skrzypiec, Małgorzata Cytryńska

**Affiliations:** 1 Department of Immunobiology, Faculty of Biology and Biotechnology, Institute of Biological Sciences, Maria Curie-Skłodowska University, Akademicka 19 St., 20-033 Lublin, Poland; wojda@poczta.umcs.lublin.pl (I.W.); cytryna@poczta.umcs.lublin.pl (M.C.); 2 Department of Industrial and Environmental Microbiology, Faculty of Biology and Biotechnology, Institute of Biological Sciences, Maria Curie-Skłodowska University, Akademicka 19 St., 20-033 Lublin, Poland; adrianw2@poczta.umcs.lublin.pl; 3 Department of Analytical Biochemistry, Faculty of Biochemistry, Biophysics and Biotechnology, Jagiellonian University, Gronostajowa 7 St., 30-387 Krakow, Poland; pawel.mak@uj.edu.pl; 4 Department of Analytical Chemistry and Biochemistry, Faculty of Materials Sciences and Ceramics, AGH University of Science and Technology, Mickiewicza 30 Ave., 30-059 Krakow, Poland; piotr.suder@agh.edu.pl; 5 Analytical Laboratory, Faculty of Chemistry, Maria Curie-Skłodowska University, M.C. Skłodowska Square 5, 20-031 Lublin, Poland; krzysztof.skrzypiec@poczta.umcs.lublin.pl

**Keywords:** α-1,3-glucan, *Aspergillus niger*, *Galleria mellonella*, insect immune response, pathogen-associated molecular pattern, pattern recognition receptors, antimicrobial peptides

## Abstract

Recognition of pathogen-associated molecular patterns (PAMPs) by appropriate pattern recognition receptors (PRRs) is a key step in activating the host immune response. The role of a fungal PAMP is attributed to β-1,3-glucan. The role of α-1,3-glucan, another fungal cell wall polysaccharide, in modulating the host immune response is not clear. This work investigates the potential of α-1,3-glucan as a fungal PAMP by analyzing the humoral immune response of the greater wax moth *Galleria mellonella* to *Aspergillus niger* α-1,3-glucan. We demonstrated that 57-kDa and 61-kDa hemolymph proteins, identified as β-1,3-glucan recognition proteins, bound to *A. niger* α-1,3-glucan. Other hemolymph proteins, i.e., apolipophorin I, apolipophorin II, prophenoloxidase, phenoloxidase activating factor, arylphorin, and serine protease, were also identified among α-1,3-glucan-interacting proteins. In response to α-1,3-glucan, a 4.5-fold and 3-fold increase in the gene expression of antifungal peptides galiomicin and gallerimycin was demonstrated, respectively. The significant increase in the level of five defense peptides, including galiomicin, corresponded well with the highest antifungal activity in hemolymph. Our results indicate that *A. niger* α-1,3-glucan is recognized by the insect immune system, and immune response is triggered by this cell wall component. Thus, the role of a fungal PAMP for α-1,3-glucan can be postulated.

## 1. Introduction

The detection of pathogens by the host immune system is based on the recognition of the characteristic conservative structures of microbial cells, called pathogen-associated molecular patterns (PAMPs), by appropriate pattern recognition receptors (PRRs) [1,2]. The search for host mechanisms responsible for recognizing fungal pathogens has led to increased interest in cell wall polysaccharides. These compounds are absent in animal cells and at least some of them are common to all fungal species. Beta-1,3-glucan and chitin, both present in hyphae and in conidia, constitute the core of the cell wall in filamentous fungi [3,4,5]. Alpha-1,3-glucan is another cell wall polysaccharide detected in many species of the class Ascomycetes and Basidiomycetes, including pathogenic *Aspergillus*, *Histoplasma*, *Blastomyces*, *Cryptococcus*, *Pneumocystis*, and *Coccidioides*. It accounts for about 46.5%, 34.5%, and 9% of the cell wall dry weight in *Histoplasma capsulatum*, *Blastomyces dermatitidis*, and *Aspergillus niger*, respectively [6,7]. The presence of α-1,3-glucan in the cell walls of entomopathogenic fungi, e.g., *Metarhizium acridum*, *Fusarium* spp., and *Aspergillus flavus*, has also been confirmed [8,9,10,11,12]. While β-1,3-glucan and chitin are the main components of the inner layer of the cell walls, α-1,3-glucan is a key component of the cell wall outer layer [13,14,15,16,17,18].

Alpha-1,3-glucan contained in the fungal cell wall has numerous structural and mechanical functions, serves as spare material, and is an important virulence factor in some pathogenic fungi [19,20,21]. For instance, α-1,3-glucan in *B. dermatitidis*, *H. capsulatum*, and *Paracoccidioides brasiliensis* is described as a molecule whose presence in the fungal cell wall protects against recognition by the host’s immune system through masking molecules considered as PAMPs, mainly β-1,3-glucan [22,23,24,25]. Deprivation of α-1,3-glucan resulted in efficient elimination of *H. capsulatum* by immunocompetent host cells [26,27,28]. A *Cryptococcus neoformans* mutant (α-1,3-glucan deficient) was incapable of infecting mice in the animal model [29,30]. A *B. dermatitidis* strain with 10,000-fold lower virulence than that of a virulent strain was almost completely devoid of cell wall α-1,3-glucan [22]. In *P. brasiliensis*, higher levels of α-1,3-glucan in the cell wall also correlated with increased virulence [20,31]. An α-1,3-glucan-deficient *A. fumigatus* strain showed a lower virulence level in the course of aspergillosis in mice, as the host’s immune system recognized and killed its cells rapidly [32]. The absence of α-1,3-glucan in the cell wall of *A. fumigatus* and *A. nidulans* increased the sensitivity of fungal cells to the action of lytic enzymes [33].

Despite the data mentioned above, the role of α-1,3-glucan in the modulation of immune response is not entirely clear. A few reports point to the immunomodulatory nature of α-1,3-glucan in mammals. Alpha-1,3-glucan isolated from different fungi can be used as an immunomodulating factor during cancer treatment [34,35]. In the chemically modified form, carboxymethylated α-1,3-glucans exhibit strong antitumor activity [36,37]. Bozza et al. [38] demonstrated that α-1,3-glucan was a potent inducer of anti-*A. fumigatus* Th1 response, indicating that this fungal cell wall component is sensed by the host immune system. Moreover, they showed that no evasion resulted from replacement of β-1,3-glucan by α-1,3-glucan on the *A. fumigatus* cell wall surface [38].

The insect immune response relies on innate humoral and cellular mechanisms. The cellular reactions include phagocytosis, nodulation, and encapsulation carried out by hemocytes. The humoral immune response consists in activation of the prophenoloxidase system and melanin formation, coagulation of hemolymph, and synthesis of antimicrobial peptides and proteins. Activation of these mechanisms requires proper recognition of non-self molecules [1,2]. Among the well-known PAMPs recognized by the insect immune system, there are components of the bacterial cell wall, i.e., peptidoglycan (PG), lipoteichoic acids (LTAs), and lipopolysaccharide (LPS), and constituents of the fungal cell wall, i.e., β-1,3-glucan [39]. In insects, the members of the β-1,3-glucan recognition protein/Gram-negative bacteria binding protein (βGRP/GNBP) family are involved in the recognition of fungal infection [40]. In most cases, genes encoding GNBPs are expressed in insect tissues involved in the immune response, e.g., the fat body and hemocytes [41,42]. The GNBPs were identified for the first time in the hemolymph of *Bombyx mori* (Lepidoptera). In insects representing various orders, homologous proteins are responsible for binding β-1,3-glucan. In *Drosophila melanogaster* (Diptera), it is GNBP3, which is very similar to the βGRPs found in lepidopteran species [42,43,44]. The amino acid sequence identity between GNBP3 from *D. melanogaster* GNBP3_DROME (NP_523986.2) and β-glucan binding protein from *B. mori* BGBP_BOMMO (NP_001036840.1) and βGRP from *G. mellonella* (CAK22401.1) is 39.76% and 40.67%, respectively (according to the NCBI database).

Since the mechanisms of innate immunity of mammals and insects show a high degree of structural and functional similarity, insect model organisms are often used in the research on fungal pathogens and the immune response to their cell wall components. The greater wax moth *Galleria mellonella* has been widely used in studies on fungal virulence, including research on *A. fumigatus*, *H. capsulatum*, *C. neoformans*, *Candida albicans*, and *Paracoccidioides lutzii* and in studies of the immune response to fungal β-1,3-glucan [45,46,47,48,49]. As shown by proteomic and transcriptomic analyses, in response to pathogen recognition, *G. mellonella* can produce a number of proteins and peptides with antimicrobial activity (AMPs), including cecropins, defensins, gallerimycin, gloverins, moricins, proline-rich peptides, anionic antimicrobial peptides, lysozyme, apolipophorin III (apoLp-III), and inhibitors of microbial proteases [50,51,52,53,54,55].

Since *G. mellonella* is widely used as a model host in studies on host-pathogen interactions, we used this insect model to answer the question whether fungal α-1,3-glucan can elicit innate immune response. Our previous report indicated that *A. niger* α-1,3-glucan can act as a virulence factor by inhibition of phenoloxidase activity in the greater wax moth hemolymph [56]. Recently, we have also demonstrated activation of *G. mellonella* cellular immune reactions by *A. niger* α-1,3-glucan, which evoked changes in the hemocytogram and formation of nodules, suggesting that the insect immune system can recognize this cell wall component of fungi [57]. This work investigates the potential role of α-1,3-glucan as a fungal PAMP by analyzing the humoral immune response of *G. mellonella* to this polysaccharide isolated from the *A. niger* cell wall, including expression of AMP-encoding genes, identification of AMPs in hemolymph, and identification of α-1,3-glucan-binding hemolymph proteins. Analyses of *G. mellonella* humoral immune response to β-1,3/1,6-glucan (laminarin) and *A. niger* conidia were carried out as a reference study.

## 2. Results

### 2.1. Survival Experiments

**Figure 1:**
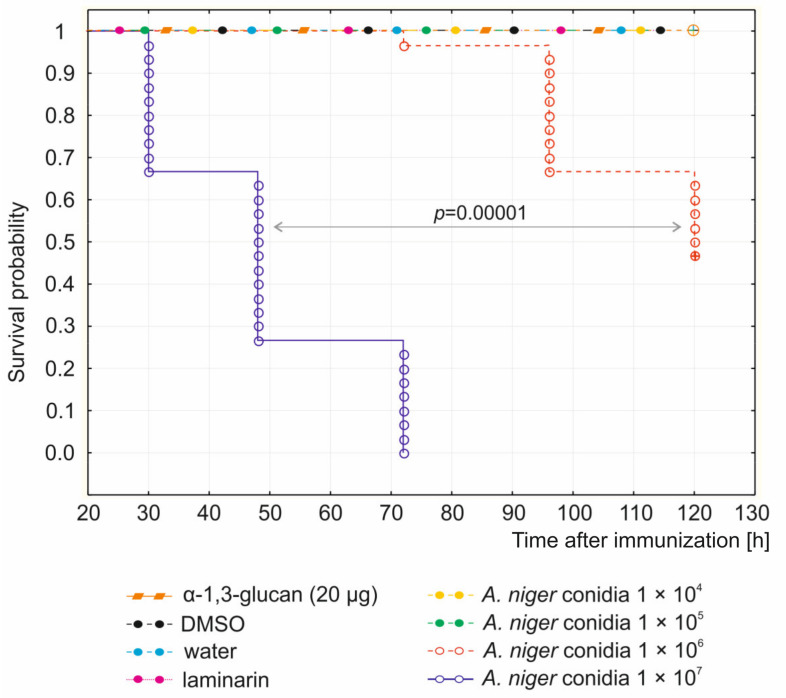
Survival analysis of *G. mellonella* larvae treated with *A. niger* α-1,3-glucan, *A. niger* conidia, or laminarin. The larvae (30 larvae per group) were injected with *A. niger* α-1,3-glucan (doses 1 µg, 5 µg, 10 µg, 20 µg), and indicated doses of *A. niger* conidia or laminarin (50 μg). The control larvae were injected with DMSO or water as described in the Materials and Methods. The probability of survival was estimated with the Kaplan–Meier method with the log-rank test. *A. niger* α-1,3-glucan in every dose used had no effect on larval survival; hence, only the survival probability after the highest dose (20 µg) used is presented.

**Figure 2:**
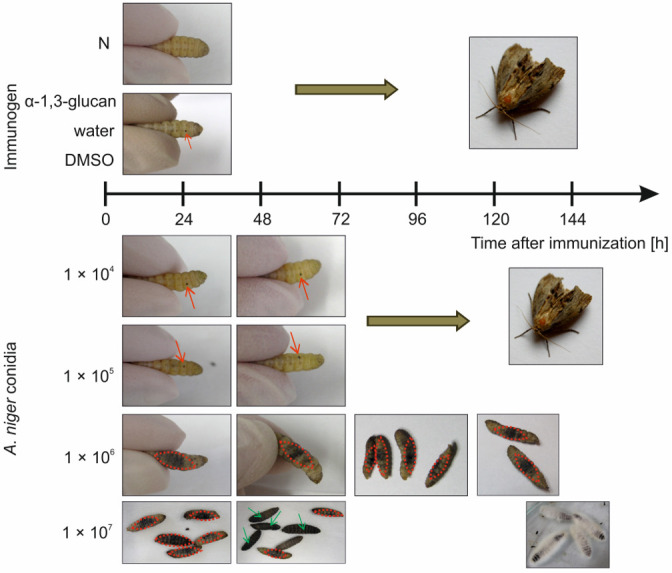
Changes in the appearance of *G. mellonella* larvae observed macroscopically after administration of *A. niger* α-1,3-glucan or different doses of *A. niger* conidia. N—Non-immunized larvae. The red arrows and the red dotted line indicate melanized spots at the injection site and melanization on the abdominal surface of the larvae, respectively. The green arrows indicate completely melanized larvae.

*A. niger* α-1,3-glucan, even in the highest dose used, i.e., 20 µg per larva, did not induce disturbed development or death of the larvae. The administration of laminarin as well as DMSO and water did not interfere with insect development either (Figure 1). After immunization, only a small dark spot at the puncture site was visible on the larva’s body (Figure 2). The number of emerging imagines corresponded to the number of caterpillars used in these experimental groups. Similarly, the doses of 1 × 10^4^ and 1 × 10^5^ of *A. niger* conidia did not have an adverse effect on the development of the immunized insects (Figure 1). In contrast, the larvae immunized with the conidial doses of 1 × 10^6^ and 1 × 10^7^ initially showed considerable darkening on the abdominal side, which then spread to the whole body (Figure 2). The survival analysis showed that the probability that larvae injected with conidia at the dose of 1 × 10^6^ would survive 120 h was less than 50%. All the larvae immunized with the dose of 1 × 10^7^ conidia were dead after 72 h. At 144 h after immunization, larvae overgrown with *A. niger* mycelium were observed (Figure 2). A statistically significant difference (*p* = 0.00001) between the survival curves determined for the larvae immunized with *A. niger* conidia at the dose 1 × 10^6^ and 1 × 10^7^ was demonstrated using the Kaplan-Meier method and the log-rank test (Figure 1). Based on the results obtained, the dose of 1 × 10^5^ *A. niger* conidia was selected for further studies as the highest one that did not disturb the development of *G. mellonella* and did not decrease larval survival.

### 2.2. Analysis of Antimicrobial Activity in G. mellonella Hemolymph after Immunization with α-1,3-Glucan

**Figure 3:**
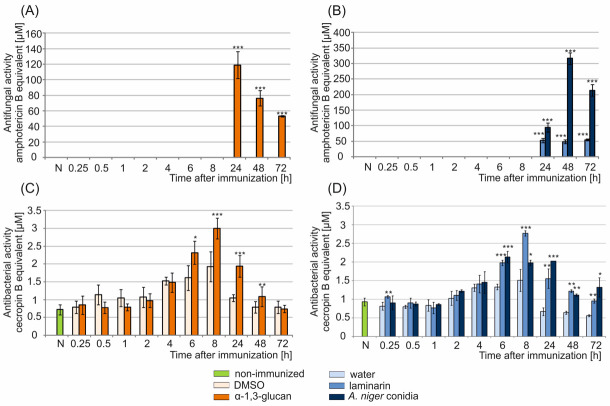
Antimicrobial activity in the hemolymph of *G. mellonella* larvae treated with *A. niger* α-1,3-glucan (**A**,**C**), *A. niger* conidia, or laminarin (**B**,**D**). The larvae were injected with *A. niger* α-1,3-glucan (5 µg), *A. niger* conidia (1 × 10^5^), or laminarin (50 µg). The control larvae were injected with DMSO or water as described in the Materials and Methods. The hemolymph was collected at the indicated time points and antifungal (**A**,**B**) and antibacterial (**C**,**D**) activity was detected on solid agar plates against *A. niger* and *E. coli*, respectively. The antifungal and antibacterial activity was expressed as amphotericin B and cecropin B equivalents, respectively. N—non-immunized larvae. Statistically significant differences between the mean values (from three independent experiments) obtained at the respective times after immunization with α-1,3-glucan and DMSO or after immunization with conidia and water are marked with asterisks: * *p* < 0.05, ** *p* ≤ 0.01, *** *p* ≤ 0.001.

Our preliminary experiments indicated that 5 µg of α-1,3-glucan used per larva was the lowest dose that triggered antifungal activity in the hemolymph. Therefore, this dose was used in further studies. The highest antifungal activity in the hemolymph of the α-1,3-glucan-challenged larvae was found 24 h post-treatment. It corresponded to the activity of 119 µM amphotericin B solution. No antifungal activity was detected in the hemolymph of non-immunized insects or at any of the time points after injection with 30% DMSO (Figure 3A). In contrast to the α-1,3-glucan treatment, the maximum antifungal activity after administration of *A. niger* conidia (1 × 10^5^) was found in the hemolymph collected 48 h post-treatment (Figure 3B), and corresponded to the activity of 316 μM amphotericin B solution. At this time point, the activity was over 4 times and over 6 times higher than after immunization with α-1,3-glucan (5 µg) and laminarin (50 µg), respectively. Interestingly, after administration of 5 μg laminarin at the dose corresponding to the applied dose of α-1,3-glucan, no antifungal activity was detected in the *G. mellonella* hemolymph (Figure 3B). The antifungal activity appeared in the hemolymph in response to a 10-fold higher laminarin dose, i.e., 50 µg. It was detected 24 h post-treatment and persisted until 72 h at the same level corresponding to the activity of approx. 50 μM amphotericin B solution, which was about 2.5-fold lower than the activity induced by injection of 5 µg of α-1,3-glucan (Figure 3A,B).

In addition to antifungal activity, antibacterial activity was detected in the hemolymph of the tested larvae (Figure 3C,D). However, in contrast to antifungal activity, immunization with both *A. niger* α-1,3-glucan and DMSO resulted in an increase in the anti-*E. coli* activity in the hemolymph (Figure 3C). A statistically significant difference in the level of antibacterial activity between the hemolymph of the α-1,3-glucan- and DMSO-injected larvae was detected 6–48 h after immunization. The maximum anti-*E. coli* activity was found 8 h after immunization with both α-1,3-glucan and DMSO. After polysaccharide administration, the activity corresponded to the activity of 3 µM cecropin B solution and was about 1.5-fold higher than after DMSO treatment (Figure 3C). However, the biggest difference in the antibacterial activity between the hemolymph of insects immunized with α-1,3-glucan and DMSO was found 24 h after administration. The level of anti-*E. coli* activity after administration of α-1,3-glucan and DMSO was equal to the activity of 1.9 μM and 1 μM cecropin B solution, respectively.

Antibacterial activity was also detected in the hemolymph of larvae immunized with laminarin and *A. niger* conidia (Figure 3D). A similarity in the kinetics of the increase in the anti-*E. coli* activity in the hemolymph of insects immunized with α-1,3-glucan (5 µg) and laminarin (50 µg) was observed. The maximum antibacterial activity, corresponding to the activity of 2.77 µM cecropin B solution, was detected 8 h after immunization with laminarin. In the case of immunization with conidia, the highest level of activity was observed 6–24 h after the treatment. However, it was lower in comparison with the α-1,3-glucan and laminarin treatment and corresponded to the activity of a 2 µM cecropin B solution (Figure 3D). The biggest difference between the level of antibacterial activity after laminarin or conidia and water administration was noted 24 h after injection. A 2- and 3-fold higher level of activity in larvae immunized with laminarin and *A. niger* conidia, respectively, was estimated in comparison with the water-injected larvae. The level of antibacterial activity after water injection corresponded to the activity of 0.74 µM cecropin B solution (Figure 3D). Bioautography analysis indicated that the activity against *E. coli* was connected with the presence of compounds with molecular weight below 6.5 kDa corresponding to antimicrobial peptides (Supplementary Materials, Figure S3).

### 2.3. Identification of Antimicrobial Peptides in G. mellonella Hemolymph

**Figure 4:**
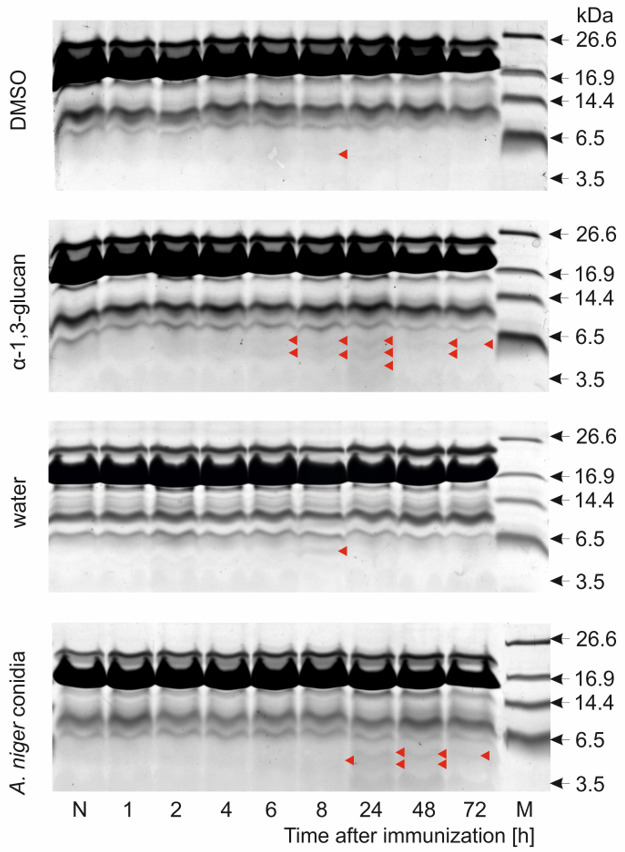
Protein/peptide profiles of hemolymph methanolic extracts of *G. mellonella* larvae treated with *A. niger* α-1,3-glucan or conidia. The larvae were injected with *A. niger* α-1,3-glucan (5 µg) or *A. niger* conidia (1 × 10^5^). The control larvae were injected with DMSO or water, respectively, as described in the Materials and Methods. The hemolymph was collected at the indicated time points; the methanolic extracts were obtained and separated by Tris-tricine SDS-PAGE. N—Non-immunized larvae; M—Molecular mass markers. Peptide bands appearing in the hemolymph after immunization are indicated with red arrowheads.

First, electrophoretic separation of hemolymph methanolic extracts containing proteins with a molecular weight below 30 kDa and peptides was carried out (Figure 4). Analysis of these profiles showed the presence of peptide bands below 6.5 kDa, which appeared 6 h after immunization with α-1,3-glucan and were present in the hemolymph up to 72 h after treatment. The changes observed in the peptide profile after injection of *A. niger* conidia also involved additional peptide bands that appeared 8 h to 72 h after immunization. The changes in the peptide profile in the hemolymph of the *A. niger* α-1,3-glucan- and conidia-challenged larvae correlated well with the activity against *A. niger* detected in the hemolymph. The analysis of the peptide profile of methanolic extracts of hemolymph from the DMSO- and water-injected larvae showed only an additional single peptide band of low intensity 8 h post-treatment (Figure 4). The profile of the peptide bands in the hemolymph after immunization of larvae with 50 μg of laminarin resembled the profile obtained after administration of 5 μg of α-1,3-glucan (Supplementary Materials, Figure S4). The injection of a lower dose of laminarin (5 µg), equal to the dose of α-1,3-glucan used, also resulted in appearance of bands corresponding to AMPs, mainly 8 h to 24 h post-treatment. However, considering the fact that no antifungal activity in the hemolymph was detected after immunization of larvae with the 5-µg dose of laminarin, the additional peptide bands most likely represented antibacterial peptides (Supplementary Materials, Figure S4).

**Figure 5:**
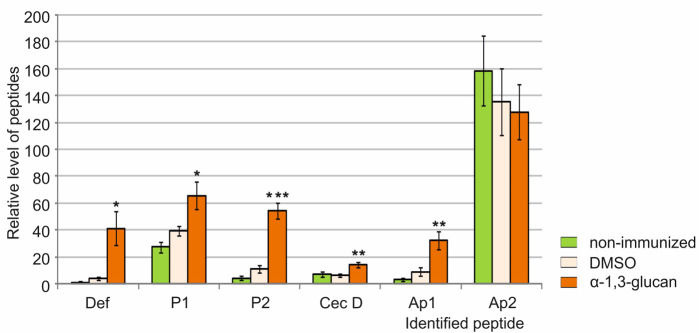
Relative level of defense peptides identified in the hemolymph of *G. mellonella* larvae treated with *A. niger* α-1,3-glucan. The larvae were injected with DMSO (control) or α-1,3-glucan (5 µg) and hemolymph was collected 24 h post-treatment. The peptide levels were determined after separation by HPLC and identified via sequencing by Edman degradation as described in the Materials and Methods. Statistical significance was determined for the differences between the mean values (from three independent experiments) for α-1,3-glucan and DMSO and marked with asterisks: * *p* < 0.05, ** *p* ≤ 0.01, *** *p* ≤ 0.001. Def—*Galleria* defensin (galiomicin), P1—Proline-rich peptide 1, P2—Proline-rich peptide 2, Cec D—Cecropin D, Ap1—Anionic peptide 1, Ap2—Anionic peptide 2.

The results presented here indicated that the AMPs were responsible for the antifungal activity in *G. mellonella* hemolymph after administration of *A. niger* α-1,3-glucan. To identify these defense peptides, methanolic extracts of hemolymph collected from larvae immunized with α-1,3-glucan and DMSO were separated by HPLC and their amino acid sequences were determined from the N-terminus. Six defense peptides were identified in the hemolymph extracts: galiomicin (*G. mellonella* defensin), proline-rich peptide 1, proline-rich peptide 2, cecropin D-like peptide, anionic peptide 1, and anionic peptide 2. The relative level of five of the six identified antimicrobial peptides in the hemolymph after administration of *A. niger*-1,3-glucan increased significantly compared to the hemolymph of the DMSO-injected larvae (Figure 5). There was a 10-fold increase in the galiomicin level, an almost 5-fold increase in the proline-rich peptide 2 level, an over 3.5-fold increase in the anionic peptide 1 level, a 2-fold increase in the cecropin level, and a 1.6-fold increase in the level of proline-rich peptide 1. The level of anionic peptide 2 did not change statistically significantly (Figure 5).

### 2.4. Analysis of Antimicrobial Peptide Gene Expression in G. mellonella Fat Body

**Figure 6:**
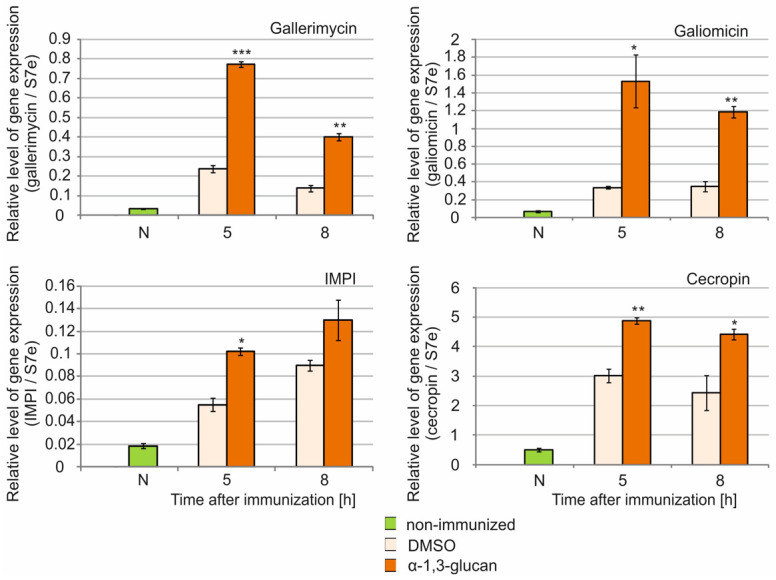
Expression of genes of selected defense peptides in the fat body of *G. mellonella* larvae after immunization with *A. niger* α-1,3-glucan. The level of expression of gallerimycin, galiomicin, IMPI, and cecropin genes was tested in the fat body of non-immunized larvae (N) and 5 h and 8 h after immunization with α-1,3-glucan (5 μg) and DMSO (control) using real time qPCR as described in the Materials and Methods. Statistically significant differences between the mean values (from three independent experiments) obtained at the respective times after immunization with α-1,3-glucan and DMSO are marked with asterisks: * *p* < 0.05, ** *p* ≤ 0.01, *** *p* ≤ 0.001.

The effect of immunization of *G. mellonella* larvae with *A. niger* α-1,3-glucan and conidia on the expression of selected AMP genes was investigated. The level of expression of genes encoding four peptides was analyzed: galiomicin (*Galleria* defensin), gallerimycin, cecropin, and insect metalloproteinase inhibitor (IMPI). The number of transcripts of genes encoding antifungal peptides, i.e., gallerimycin and galiomicin, increased more than 3- and 4.5-fold, respectively, 5 h after immunization of the larvae with α-1,3-glucan, compared to the DMSO-injected larvae (Figure 6). At 8 h after immunization, a decrease in the number of transcripts of these genes by 12% and 26%, respectively, was observed. The level of cecropin gene expression was 1.6- and 1.8-fold higher after administration of α-1,3-glucan relative to DMSO administration, 5 h and 8 h after treatment, respectively. The level of IMPI gene transcripts increased 1.9- and 1.4-fold after immunization with α-1,3-glucan compared to DMSO administration 5 h and 8 h post-treatment, respectively. Therefore, the highest level of expression of the three examined genes was found 5 h after the administration of *A. niger* α-1,3-glucan. The expression level decreased 8 h after immunization (Figure 6).

**Figure 7:**
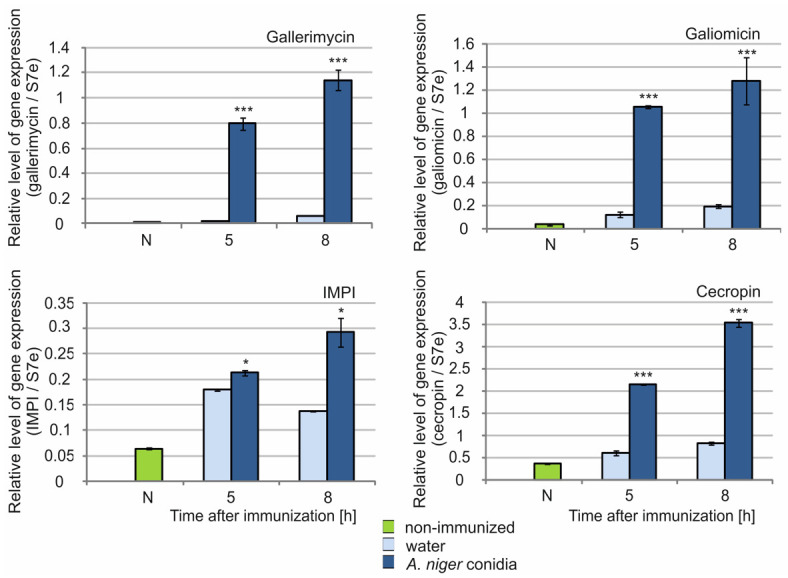
Expression of genes of selected defense peptides in the fat body of *G. mellonella* larvae after immunization with *A. niger* conidia. The level of expression of gallerimycin, galiomicin, IMPI, and cecropin genes was tested in the fat body of non-immunized larvae (N) and 5 h and 8 h after immunization with *A. niger* conidia (1 × 10^5^) and water (control) using real time qPCR as described in the Materials and Methods. Statistically significant differences between the mean values obtained at the respective times after immunization with conidia and water are marked with asterisks: * *p* < 0.05, *** *p* ≤ 0.001.

The administration of *A. niger* conidia (1 × 10^5^) resulted in a 33-fold increase in gallerimycin gene expression relative to the level after injection of water within 5 h after the treatment (Figure 7). In the longer term, the expression level was still relatively high, i.e., 18-fold higher than the level detected in the water-injected larvae. The highest level of galiomicin gene expression was also observed 5 h after conidia administration. There was an 8.7- and 6.7-fold increase in the level of expression of this gene relative to the level in the water-injected caterpillars 5 h and 8 h after treatment, respectively. The expression of the cecropin-encoding gene increased 3.5- and 4.3-fold during 5 h and 8 h, respectively, compared to the level after water administration. The number of transcripts of the IMPI gene increased by about 1.3- and 2-fold during 5 h and 8 h, respectively, after immunization with *A. niger* conidia compared to water administration (Figure 7).

### 2.5. Identification of G. mellonella Hemolymph Proteins Binding to A. niger α-1,3-Glucan

**Figure 8:**
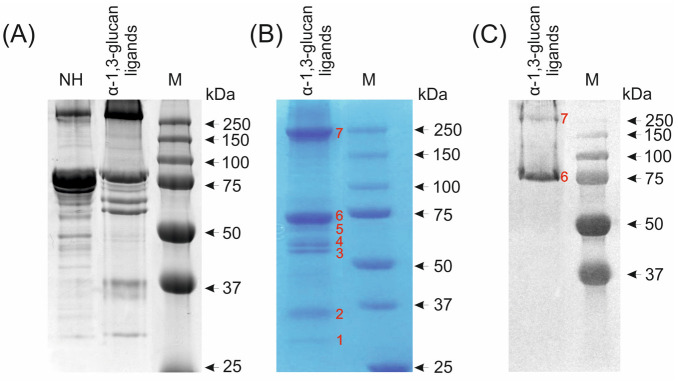
Electrophoretic separation (**A**,**B**) and immunodetection (**C**) of *G. mellonella A. niger* α-1,3-glucan-binding hemolymph proteins. The hemolymph of non-immunized larvae (NH) was incubated with the α-1,3-glucan suspension for 0.5 h. Hemolymph proteins that bound to α-1,3-glucan (α-1,3-glucan ligands) were separated by SDS-PAGE and stained with Coomassie Brilliant Blue R-250 (**A**) or transferred to a PVDF membrane and stained with Coomassie Brilliant Blue R-250 (**B**) or immunoblotted (**C**) with antibodies against apoLp-I/II. Protein bands marked with red numbers 1 to 5 on the membrane (**B**) were excised from the membrane and the proteins were sequenced from the N-terminus. Proteins in lanes 6 and 7 (**B**) were identified by mass spectrometry and by immunoblotting using antibodies against apoLp-I/II (**C**). M—Molecular mass markers.

Hemolymph proteins that bound to α-1,3-glucan were electrophoretically separated, visualized on polyacrylamide gel (Figure 8A), and transferred onto a membrane. After staining, 7 protein bands (marked with numbers from 1 to 7) were selected and designed for further identification (Figure 8B). Edman degradation sequencing allowed identification of five of them, marked with numbers from 1 to 5 (Figure 8, Table 1). The 30-kDa protein (1) was identified as a factor activating *G. mellonella* phenoloxidase; it also has significant similarities to the serine peptidases of various lepidopteran species. The 37-kDa protein (2) is similar to the *Papilio polytes* tryptase and other serine peptidases of butterflies and fully corresponds to the protein encoded in the genome of *G. mellonella* whose role has not yet been described. The 55-kDa protein (3) was identified as *G. mellonella* prophenoloxidase. Proteins of molecular weight 57 kDa (4) and 61 kDa (5) were identified as *G. mellonella* β-1,3-glucan-binding proteins, showing significant similarity to β-1,3-glucan-binding proteins of other lepidopteran species (Table 1). To identify proteins in bands (6) and (7) with molecular weight of 75 kDa and 250 kDa, respectively, immunoblotting with antibodies directed against apoLp-I/II was used (Figure 8C) [58]. These proteins were also analyzed by mass spectrometry, which confirmed the results obtained using the antibodies. In addition, *G. mellonella* arylphorin was identified in the band corresponding to the 75 kDa molecular weight. It was also found that the 250-kDa protein was similar to *Plutella xylostella* and *Helicoverpa armigera* lipophorin (Table 1).

## 3. Discussion

Recognition of the PAMP structures of pathogen cells is a key step in activating immune response. The role of the molecular determinant of fungal cells recognized by the insect immune system is most often attributed to the β-1,3-glucan molecule [59]. This polysaccharide is detected by β-1,3-glucan recognition proteins (βGRPs/GNBPs) [60,61]. Receptors of this type have been identified, e.g., in *B. mori, D. melanogaster, G. mellonella, Manduca sexta, Plodia interpuncella, Tenebrio molitor*, and *Thitarodes pui* [44,62,63,64].

Our results showed that two 57-kDa and 61-kDa proteins present in the hemolymph of naive *G. mellonella* larvae identified as β-1,3-glucan recognition proteins bound to *A. niger* α-1,3-glucan. These proteins exhibit 95% similarity to the βGRPs of other members of the Lepidoptera order, such as *Papilio xuthus* and *P. interpunctella*. These receptors are likely to be involved in recognition of *A. niger* α-1,3-glucan by the *G. mellonella* immune system. Other hemolymph proteins that interacted with *A. niger* α-1,3-glucan were also identified, i.e., apoLp-I, apoLp-II, prophenoloxidase, phenoloxidase activating factor, arylphorin, and serine protease similar to peptidases from other representatives of the Lepidoptera order. It has been shown that the activity of the phenoloxidase system, one of the first mechanisms triggered in response to infection, is inhibited in the hemolymph of *G. mellonella* larvae within a short time after immunization with *A. niger* α-1,3-glucan [56], probably due to binding of prophenoloxidase and serine proteases involved in its activation to the polysaccharide, as demonstrated in this work. Interestingly, the present study showed that *G. mellonella* apoLp-III did not bind to fungal α-1,3-glucan. This was surprising because apoLp-III is considered as a PRR molecule capable of interacting with various PAMPs, e.g., LTAs, LPS and β-1,3-glucan, which are components of the cell walls of Gram-positive and Gram-negative bacteria and fungi. The ability of apoLp-III to bind to the conidia of different filamentous fungi, including *A. niger*, yeast cells, as well as cells of different Gram-positive and Gram-negative bacteria has been demonstrated [65,66,67,68,69,70,71].

The results of our experiments revealed that, in response to *A. niger* α-1,3-glucan, the antifungal response mechanisms were activated, which was manifested by an increase in the expression of the genes encoding antifungal peptides galiomicin and gallerimycin. Galiomicin has in vitro activity against a number of filamentous fungi, including *A. niger, F. oxysporum, Pyricularia grisea*, *Trichoderma harzianum,* and *T. viride,* and yeasts *C. albicans* and *C. neoformans*. Gallerimycin is active against filamentous fungi, e.g., *Metarhizium anispoliae* (*robertsii*) but not yeast *Saccharomyces cerevisiae* [52,54,72,73,74]. The study demonstrated a 4.5-fold and 3-fold increase in the galiomicin gene expression and gallerimycin gene expression, respectively, 5 h after immunization of *G. mellonella* larvae with α-1,3-glucan. After immunization with *A. niger* conidia, a 8.7-fold and 33-fold increase in the galiomicin and gallerimycin gene expression, respectively, was found. According to the literature data, infection of *G. mellonella* larvae with the entomopathogenic fungus *Beauveria bassiana* also led to increased expression of genes encoding galiomicin and gallerimycin. The highest level of expression of these two genes was 3- and 5-fold higher, respectively, than the expression level in non-immunized larvae [75]. An increase in the gallerimycin gene expression was also noted in *G. mellonella* larvae immunized with a mixture of *E. coli* and *S. cerevisiae* [76]. High levels of galiomicin and gallerimycin gene expression were found after *G. mellonella* immunization with different species of *Candida*, they were positively correlated with fungal virulence. A higher level of expression was noted after administration of highly virulent *C. albicans*, in comparison with less virulent *C. dubliniensis* [51,55,77,78].

The level of expression of the cecropin gene analyzed in this study increased 1.6-fold and 1.8-fold, respectively, 5 h and 8 h after immunization of larvae with α-1,3-glucan. In turn, it increased 3.5- and 4.3-fold at 5 h and 8 h post-treatment, respectively, after immunization with *A. niger* conidia. Although cecropins are mainly regarded as antibacterial peptides, antifungal activity has also been attributed to some cecropins, e.g., in *D. melanogaster*, *B. mori*, or *G. mellonella* [52,79,80,81].

In addition to antimicrobial peptides, the insect’s body uses other peptides to help counteract the infection. The activity of microbial proteases may be limited by the action of an insect metalloproteinase inhibitor (IMPI) which can inhibit *M. robertsii* spore germination in vitro [82]. Our results indicated that the level of the IMPI gene expression increased approx. 2 times in the fat body of the *A. niger* α-1,3-glucan- and conidia-challenged larvae. Vertyporokh and Wojda [75] showed that as a result of natural infection of *G. mellonella* with *B. bassiana*, the level of IMPI transcripts increased at further time points after infection.

The induction of antifungal peptides synthesis in *D. melanogaster* (Diptera) occurs as a result of the Toll signaling pathway activation after recognition of β-1,3-glucans as fungal PAMPs by βGRPs/GNBPs. It was confirmed that also in Lepidoptera, including *M. sexta* and *B. mori*, the Toll pathway is involved in the immune response [83]. Analysis of the *G. mellonella* transcriptome revealed the presence of transcripts of many components of this pathway, including Toll receptor homologs and Spaetzle cytokine homolog [55]. Moreover, it is known that βGRP in Lepidoptera consists of two domains: N-terminal domain which plays a critical role for the detection of pathogen’s PAMPs, e.g., different types of β-glucan, and a C-terminal glucanase-like domain without glucanase activity and without affinity to the β-1,3-glucan, which is nevertheless required for the activation of Toll signaling pathway [40]. However, in order to establish the signal transduction pathway leading to the induction of the expression of antimicrobial peptide synthesis in response to the presence of fungal α-1,3-glucan, additional studies would be needed.

As a result of *A. niger* α-1,3-glucan recognition by the *G. mellonella* immune system and induction of AMP gene expression in the fat body, the hemolymph exhibited antifungal activity. Anti-*A. niger* activity was detected in the hemolymph 24 h after administration of this polysaccharide; it persisted up to 72 h after immunization. These results clearly demonstrated that *A. niger* α-1,3-glucan was a stronger inducer of antifungal activity in *G. mellonella* than β-1,3/β-1,6-glucan (laminarin) used in the study. Only a 10-fold higher dose of laminarin than that of α-1,3-glucan induced this activity, however still at a substantially lower level than after α-1,3-glucan administration. The maximum level of antifungal activity in the hemolymph of the *A. niger* conidia-challenged larvae was found 48 h post-treatment. The temporal shift of the activity peak towards the injection of α-1,3-glucan may have resulted from the coverage of conidia by a hydrophobic rodlet layer made of hydrophobins and melanin, which protects them from recognition by the immune system [84]. It is assumed that the immune response is triggered when such components as β-1,3-glucan, α-1,3-glucan, galactosaminogalactan, and galactomannan hidden under the protective layer are exposed, e.g., during spore germination. The higher level of antifungal activity in the hemolymph of the conidia-challenged larvae than in the hemolymph of the α-1,3-glucan- and laminarin-immunized insects was most likely caused by the induction of immune response by a mixture of many components found in the fungal cell wall. It is worth noting that, after the injection of water and DMSO, no antifungal activity was found in the *G. mellonella* hemolymph, in contrast to the detected antibacterial activity, which indicates specificity of this reaction. As shown in the literature, immunization of *G. mellonella* larvae with *F. oxysporum* conidia induced the appearance of antifungal activity in the hemolymph 48 h after administration, whereas immunization with *C. albicans* cells induced this activity 24 h post-treatment, however, at a substantially lower level [53]. As a result of the natural infection of *G. mellonella* with *B. bassiana*, the antifungal activity also appeared after 24 h and increased with time after immunization [85].

In addition to antifungal activity, antibacterial activity measured against *E. coli* was detected in larval hemolymph. The highest level of antibacterial activity was found in the hemolymph 8 h after the administration of α-1,3-glucan and laminarin. Immunization with *A. niger* conidia induced the activity against *E. coli* to a lesser extent. Literature data show that the maximum level of antibacterial activity appeared 24 h after administration of *E. coli* and *M. luteus* to *G. mellonella* larvae and slightly earlier, i.e., at 12–18 h after injection of *Pseudomonas aeruginosa*, depending on the tested bacterial strain [53,86,87]. The present results allow formulating a conclusion that, unlike the antifungal activity, the antibacterial activity induced in *G. mellonella* by α-1,3-glucan, conidia, and laminarin was less specific, since it was also detected after administration of DMSO and water.

The analysis of the protein-peptide profiles of the hemolymph of the α-1,3-glucan-immunized larvae revealed the presence of additional bands less than 6.5 kDa corresponding to the antimicrobial peptides. The kinetics of their appearance in the hemolymph correlated well with the antimicrobial activity of the hemolymph. It was shown that, 24 h after injection of α-1,3-glucan, the level of five of the six identified defense peptides in the hemolymph was increased, corresponding well with the highest antifungal activity. There has been a 10-fold, 5-fold, 3.5-fold, 2-fold, and 1.6-fold increase in the level of galiomicin, proline-rich peptide 2, anionic peptide 1, cecropin D, and proline-rich peptide 1, respectively. An increase in the galiomicin level in the hemolymph was also demonstrated after immunization of *G. mellonella* larvae with *F. oxysporum* conidia [53]. A several-fold increase in the level of proline-rich peptide 2 in hemolymph was also noted in *M. luteus*-, *E. coli*-, *C. albicans*-, and *F. oxysporum*-injected larvae [52,53]. It is worth noting that in vitro activity against *A. niger* was demonstrated by galiomicin, anionic peptide 1, and cecropin D [52,53,88].

The results of our study on *G. mellonella* immune response to *A. niger* α-1,3-glucan indicate a dual role of this fungal cell wall component in modulating the immune response of insects (results presented in this work; [56,57]). It has been unequivocally shown that *A. niger* α-1,3-glucan can be recognized by the *G. mellonella* immune system, which leads to the activation of humoral immune reactions, including synthesis of peptides with antifungal activity (this work), and activation of cellular immune response [57]. Therefore, next to β-1,3-glucan, it can be classified among PAMPs that are molecular determinants of fungi. On the other hand, α-1,3-glucan can be regarded as a fungal virulence factor since it is responsible for inhibiting one of the earliest insect immune responses, i.e., the activation of the phenoloxidase system [56]. Interestingly, according to the literature data, the role of fungal α-1,3-glucan in the immune response of mammals may also be twofold. On the one hand, this polysaccharide stimulated the immune response in a mouse model and ex vivo activated dendritic cells [38,89]. On the other hand, it protected *A. fumigatus* conidia from phagocytosis by murine macrophages [32,90].

The results of this study indicate that *A. niger* α-1,3-glucan is recognized by the immune system of *G. mellonella* larvae and, antifungal response is activated in response to this fungal cell wall component. Thus, the role of a fungal PAMP molecule for α-1,3-glucan can be postulated.

## 4. Materials and Methods

### 4.1. Microorganisms and Growth Conditions

The filamentous fungus *Aspergillus niger* CBS 554.65 (wild type) was received from the Centraalbureau voor Schimmelcultures (Utrecht, the Netherlands). The microorganism was grown on potato dextrose agar slants at 28 °C until conidia were obtained and then maintained at 4 °C. The composition of the medium was as described elsewhere [91].

The Gram-negative bacterium *Escherichia coli* D31 (CGSC 5165; Genetic Stock Centre, New Haven, CT, USA) [92] was grown in liquid Lysogeny Broth (LB; Biocorp, Warszawa, Poland) medium at 37 °C with shaking. The bacteria were stored at 4 °C.

### 4.2. Immunodetection of α-1,3-Glucan in A. niger

For immunodetection of α-1,3-glucan in *A. niger* cell walls, mycelium and conidiophores were fixed on microscopic 8-well glass slides (Nunc LAB-TEK slides; Thermo Scientific, Waltham, MA, USA) with 3% formaldehyde for 30 min at 65 °C [93]. After three washing steps for 2 min in phosphate buffered saline (PBS; 137 mM NaCl, 2.7 mM KCl, 8.1 mM Na_2_HPO_4_, 1.5 mM KH_2_PO_4_, pH 7.4), the material was incubated for 5 min in 1% Tween-20 in PBS and washed again with PBS. Then, 150 µL of mouse antibodies against α-1,3-glucan (final concentration 0.1 mg/mL; Sigma-Aldrich, Saint Louis, MO, USA) were added to each well and the slides were incubated for 24 h at 4 °C. Goat anti-mouse Alexa-Fluor 488 labeled antibodies were used as secondary antibodies (150 µL, 0.1 mg/mL; Sigma-Aldrich, Saint Louis, MO, USA). After 2-h incubation at 37 °C in the dark, the slides were washed three times with PBS and imaged using a laser scanning confocal microscope Olympus BX 51 (Olympus, Shinjuku, Tokyo, Japan) (excitation and emission wavelength 470/500 nm and 525/550 nm, respectively). The presence of α-1,3-glucan in the examined material was confirmed (Supplementary Materials, Figure S1).

### 4.3. Isolation and Characterization of α-1,3-Glucan from A. niger

Fresh mycelium of *A. niger* was lyophilized and milled using laboratory grinder (WŻ-1, ver. S.1, ZBPPI Instruments, Bydgoszcz, Poland). After drying, the material was used for isolation of alkali-soluble polysaccharides according to the method described by Kiho et al. [94]. Briefly, the dried material (100 g) was successively extracted with methanol, NaCl (9 g/L), hot water, Na_2_CO_3_ (50 g/L), and finally with 1 M NaOH containing 0.2 g NaBH_4_ for 24 h at room temperature. The final extract was neutralized with 1 M HCl under constant mixing, and the precipitated alkali-soluble fraction was washed with water, collected by centrifugation, and lyophilized. Structural analyses (composition analysis, methylation analysis, Fourier transform infrared spectroscopy, and nuclear magnetic resonance spectroscopy) revealed that the alkali-soluble preparation of α-glucans contained a linear polymer composed almost exclusively of (1→3)-linked α-d-glucose (92.3%). The yield of alkali-soluble α-1,3-glucan isolated from *A. niger* CBS 554.65 was 8.4% of mycelium dry mass [7]. According to the literature data, a minimum length of *A. niger* α-1,3-glucan could be estimated as approx. 330 DP (degree of polymerization), which corresponds to 53.5 kDa [95].

Before use, the lyophilized α-1,3-glucan was suspended in 30% dimethylsulfoxide (DMSO) and sonicated in order to obtain a homogenous suspension (5 × 1 min; 30-s pulse, 30 s off) with a Ultrasonic Processor XL 2020 sonicator (20 KHz Frequency, MISONIX, Farmingdale, NY, USA).

### 4.4. Atomic Force Microscopy Imaging of α-1,3-Glucan

The suspension of α-1,3-glucan in 30% DMSO or 30% DMSO alone was placed onto a mica disk and dried in a desiccator overnight. Then, the samples were imaged in NanoScope V AFM (Veeco, Plainview, NY, USA) using the PeakForce QNM operation mode and a RTESPA-300 silicon probe with spring constant 20–80 N/m (Bruker Nano Inc., Billerica, USA). Three fields 1 µm × 1 µm were imaged for each sample. The images were analyzed with NanoScope Analysis software ver. 1.40 (Veeco, Plainview, NY, USA). It was found that α-1,3-glucan intended for administration to the larvae by injection was in the form of spherical nanoparticles with a diameter of about 7 nm (±0.5) (Supplementary Materials, Figure S2).

### 4.5. Insect Rearing and Immunization

The last instar larvae (250–300 mg) of the greater wax moth *Galleria mellonella* (Lepidoptera: Pyralidae) maintained in a continuous laboratory culture were used in the study. The insects were reared on honeybee nest debris at 30 °C in the dark. The larvae were immunized by intrahemocoelic injection of 3 µL of non-pyrogenic water containing *A. niger* conidia (1 × 10^4^, 1 × 10^5^, 1 × 10^6^, 1 × 10^7^ per larva) or laminarin, i.e., β-1,3/1,6-glucan from *Laminaria digitata* (Sigma-Aldrich, Saint Louis, MO, USA) (5 µg and 50 µg per larva). For immunization with *A. niger* α-1,3-glucan, the larvae were injected with 3 µL of 30% DMSO containing 1 µg, 5 µg, 10 µg, or 20 µg of this polysaccharide. The control larvae were injected with 3 µL of non-pyrogenic water or 30% DMSO, respectively (final concentration in larval hemocoel—approx. 1.25%).

### 4.6. Survival Experiments

The larvae were injected intrahemocoelically with different doses of *A. niger* α-1,3-glucan, *A. niger* conidia, and laminarin, as well as water and 30% DMSO used as controls, as described in Section 2.5. Each group consisted of 30 individuals. The survival was checked at specific time points: 24 h, 30 h, 48 h, 72 h, 96 h, and 120 h after immunization and was completed at the time of death of the tested larvae or transformation of pupae into imagines. The entire experimental layout was repeated three times. The probability of larval survival was estimated with the Kaplan–Meier method with the log-rank test [96,97].

### 4.7. Collection of Hemolymph and Preparation of Hemolymph Methanolic Extracts

The hemolymph was collected in sterile conditions at different time points after treatment (0.25–72 h) into chilled Eppendorf tubes containing crystals of phenylthiourea (PTU) to prevent melanization, and the cell-free hemolymph was obtained as described in our previous papers [53,98]. Briefly, the caterpillars were chilled for 15 min at 4 °C and their abdominal surface was disinfected with a 70% (*v*/*v*) ethanol solution. The larval abdomen was then punctured with a sterile needle and the outflowing hemolymph was transferred to an Eppendorf tube. To obtain hemocyte-free hemolymph, the samples were centrifuged at 200× *g* for 5 min. The supernatants were centrifuged again at 20,000× *g* for 10 min at 4 °C to remove cell debris. The hemolymph samples were then kept at −20 °C.

The methanolic extracts were obtained from hemocyte-free hemolymph by 10-fold dilution with the extraction solution (methanol:glacial acetic acid:water; 90:1:9, *v*/*v*/*v*) and centrifugation at 20,000× *g* for 30 min at 4 °C. A supernatant containing proteins below 30 kDa and peptides was transferred to a new Eppendorf tube and freeze-dried [52]. The hemolymph extract was dissolved in 0.1% aqueous trifluoroacetic acid (TFA) and the lipids were then removed by mixing the extract with an equal volume of n-hexane, followed by centrifugation (20,000× *g*, 15 min, 4 °C). The upper lipid layer was removed and a volume of ethyl acetate equal to the volume of n-hexane was added to the bottom layer. After mixing and centrifugation (20,000× *g*, 15 min, 4 °C), the upper layer was removed. The lower layer was transferred to a new Eppendorf tube and lyophilized [52].

### 4.8. Antimicrobial Activity Assays

Antibacterial activity was determined using the radial diffusion assay on solid agar plates containing viable *E. coli* D31 cells and hen egg white lysozyme (EWL; 2 mg/mL) [99,100,101]. Antifungal activity was assessed with the use of the radial diffusion assay on PDA plates (5% potato extract, 0.5% dextrose, 0.7% agar; *w*/*v*) containing *A. niger* conidia (0.2 × 10^6^ conidia/mL of the medium). Appropriately diluted hemolymph samples (4 µL) were added to the wells in Petri plates, which were then incubated at 37 °C (*E. coli*) or 28 °C (*A. niger*). Microbial growth inhibition zones were measured after 24-h incubation. Antibacterial or antifungal activity was expressed as an equivalent activity of cecropin B [101] or amphotericin B [53], respectively.

Antimicrobial activity was also detected using bioautography as described previously [100]. After separation of the hemolymph samples (150 µg of total protein) by SDS-PAGE in 13.8% polyacrylamide gels, the gels were washed twice in 2.5% Triton X-100 (Bio-Rad, Hercules, CA, USA) for 20 min to remove the denaturing agent and then twice in 50 mM Tris-HCl pH 7.5 and in liquid LB medium for 15 min. In the final stage, the gels were overlaid with liquefied LB medium with 0.7% agar supplemented with EWL (2 mg/mL), streptomycin (0.14 mg/mL), and 24-h *E. coli* D31 culture. After 24-h incubation at 37 °C, clear zones were observed in the medium, indicating inhibition of bacterial growth.

### 4.9. Analysis of the Polypeptide Profile in Hemolymph

From each of the 30 larvae belonging to the experimental group, 30 µL of hemolymph were taken into the pool. Methanolic extracts were prepared from equal volumes of hemolymph from non-immunized larvae and 24 h after α-1,3-glucan immunization and DMSO administration. Freeze-dried lipid-free hemolymph methanolic extracts were dissolved in 0.1% TFA. Chromatographic separations of peptides and proteins present in the tested preparations were carried out using the Dionex P680 HPLC system (Dionex, Sunnyvale, CA, USA) and Discovery Bio Wide Pore C18 4.6 mm × 250 mm columns (Sigma-Aldrich, Saint Louis, MO, USA), using 0.1% TFA as solvent A and 0.07% TFA:80% acetonitrile as solvent B (*v*/*v*). The location of the peaks on the chromatogram allowed initial identification of antimicrobial proteins and peptides, and the assessment of their height facilitated comparative quantitative analysis thereof. After separation of these compounds by Tris-tricine SDS-PAGE and transfer to the polyvinylidene difluoride (PVDF) membrane [52,53], the identification was confirmed by determination of five amino acid residues from the N-terminus by Edman degradation using the automatic Procise 491 sequencer (Applied Biosystems, Waltham, MA, USA).

### 4.10. Analysis of Peptide Gene Expression in the Fat Body

#### 4.10.1. Collection of Fat Bodies

The collection of fat bodies was carried out in sterile conditions from 5 larvae in each group 5 h and 8 h after immunization. The larvae were cooled down and sterilized in 70% ethanol. The fat bodies were isolated under ice-cold Ringer’s solution (172 mM KCl, 68 mM NaCl, 5 mM NaHCO_3_, pH 6.1, osmolarity 420 mOsm; all ingredients of cell-culture grade). The isolated fat bodies were transferred to Eppendorf tubes containing Ringer’s solution. The liquid was then removed and the organs were frozen in liquid nitrogen for 10 min. The isolated fat bodies were kept at −70 °C for RNA isolation.

#### 4.10.2. Peptide Gene Expression

Total RNA from the fat bodies pooled from 5 larvae was isolated using a GenElute Mammalian Total RNA Extraction Kit (Sigma-Aldrich, Saint Louis, MO, USA). Treatment with DNA-se was performed with the use of the Turbo DNA-free^TM^ kit (Thermo Scientific, Waltham, MA, USA). Reverse transcription was performed using 1 µg of total RNA and random hexamer primers (High Capacity cDNA Reverse Transcription Kit; Life Technologies, Carlsbad, CA, USA). Quantitative PCR from the cDNA obtained was performed using a Step One Plus PCR System (Applied Biosystems, Waltham, MA, USA). The primers for S7e, gallerimycin, cecropin, galiomicin, and insect metalloproteinase inhibitor (IMPI) genes used in the study were described previously [102,103]. The real time qPCR conditions were 95 °C 10 min, 44 × (95 °C, 15 s—denaturation; 60 °C, 1 min—annealing and extension). As a standard curve, PCR amplification was performed with several dilutions of the DNA template from the immunized larvae. The amount of mRNA detected was normalized to ribosomal protein S7e mRNA.

### 4.11. Identification of α-1,3-Glucan Ligands in Insect Hemolymph

#### 4.11.1. Binding of Hemolymph Proteins to α-1,3-Glucan

To determine which *G. mellonella* hemolymph proteins are capable of binding to α-1,3-glucan, the polysaccharide suspension was incubated with hemolymph obtained from the non-immunized larvae. In each of 75 Eppendorf tubes, a suspension containing 100 µg of α-1,3-glucan in 30% DMSO was mixed with the hemolymph (300 µg of total proteins) in a ratio of 3:2 (*v*/*v*). The mixture was incubated for 30 min at 4 °C allowing hemolymph proteins to bind to α-1,3-glucan. The contents of the tubes were centrifuged (10,000× *g*, 5 min). The pellets of each of the two tubes were suspended in 80 µL of 1% Triton X-100, thus reducing the number of tubes by a half, and incubated for 5 min to remove non-specifically bound proteins. The samples were then centrifuged (10,000× *g*, 5 min) and the pellets were washed three times in 200 µL PBS buffer. Finally, the pellets in three test tubes were suspended in sample buffer [104] and subjected to further analyses, as described in Section 4.11.2 and Section 4.11.3.

#### 4.11.2. Identification of Proteins by N-terminal Sequencing

The samples obtained as described in Section 4.11.1 were heated at 98 °C for 8 min and centrifuged (20,000× *g*, 15 min). The supernatants were pooled and filtered using PVDF centrifuge tubes with a 0.45 µm pore diameter (12,000× *g*, 4 min). The samples were then heated at 98 °C until the volume was reduced to 20 μL and subjected to SDS-PAGE in 13.8% polyacrylamide gel. The gels were then placed in a transfer buffer (5 mM CAPS, 10% methanol, pH 11) and protein transfer to the PVDF membrane was carried out. The membranes were washed with water and then with methanol, stained in 0.1% Coomassie Brilliant Blue R-250 (in 40% methanol and 10% acetic acid) for 1 min, and then destained in 50% methanol, washed with deionized water, and dried. Protein sequencing by Edman degradation was carried out using the automatic Procise 491 sequencer (Applied Biosystems, Waltham, MA, USA) after excision of stained protein bands from the membrane, and the resulting sequences were compared to peptide and protein sequences found in the National Center for Biotechnology Information (NCBI) *Galleria mellonella* Annotation Release 100 database (Software version 8.1).

#### 4.11.3. Identification Using Immunodetection with Specific Antibodies

In order to check whether apolipophorin I and II (apoLp-I/II) bind to α-1,3-glucan, immunoblotting with antibodies directed against these proteins was performed. Proteins that bound to α-1,3-glucan were separated by SDS-PAGE in 7.5% polyacrylamide gel and transferred to the PVDF membrane. The membranes were incubated for 1 h in Tris-buffered saline (TBS; 10 mM Tris-HCl pH 7.4, 0.9% NaCl) with 5% non-fat milk powder and then incubated for 1 h with rabbit antibodies directed against *B. mori* apoLp-I/II (1:3000; kindly gifted by Prof. Chikara Kaito, Laboratory of Microbiology, Graduate School of Pharmaceutical Sciences, University of Tokyo, Japan) in TBS with 5% non-fat milk powder. The membranes were washed three times for 10 min in TBS with 1% Triton X-100 and then incubated for 1 h with secondary goat anti-rabbit alkaline phosphatase-conjugated antibodies (1:30,000; Sigma-Aldrich, Saint Louis, MO, USA) diluted in TBS with 5% non-fat milk powder. After two washing steps in TBS buffer for 10 min and another 10 min in AP buffer (100 mM Tris-HCl pH 9.5, 100 mM NaCl, 5 mM MgCl_2_), bands corresponding to recognized proteins were visualized using a mixture of nitro blue tetrazolium (NBT; 0.9 mg/mL in 70% aqueous dimethylformamide DMF) and 5-bromo-4-chloro-indolyl-phosphate (BCIP; 4.5 mg/mL in DMF) [105].

#### 4.11.4. Mass Spectrometry

For identification of proteins by mass spectrometry, their bands were cut out from the Coomassie Brilliant Blue stained gels and destained by covering with 100 μL of 50 mM ammonium bicarbonate NH_4_HCO_3_ (pH 7.5) and shaking 5 min at 37 °C. Then, acetonitrile (ACN, gradient grade; JT Baker, Deventer, the Netherlands) in a volume of 100 μL was added and the samples were incubated for additional 5 min. Next, the solution was replaced with 100–200 μL of ACN (depending on the band volume) and left for complete dehydration. The procedure was repeated at least 2–3 times, depending on the staining intensity of the bands. Reduction of disulfide bonds was carried out using 50 mM dithiotreitol (DTT, stock solution 10x) in 50 mM NH_4_HCO_3_, pH 7.5. The process was continued for 5 min at 90 °C with shaking. Then, iodoacetamide (50 mM in an NH_4_HCO_3_ solution) was added to a final concentration of 5 mM and incubated for 5 min at 90 °C in darkness. After the reduction/alkylation process, residual reagents were washed out with 50% (*v*/*v*) ACN in 25 mM NH_4_HCO_3_ buffer. The gel pieces were then dehydrated in 100% ACN and dried in a vacuum dryer for 15 min. Next, they were incubated in 15 µL of a trypsin solution (10 ng/µL in 25 mM NH_4_HCO_3_, pH 8.0; Gold, mass spectrometry grade, Promega, Madison, WI, USA) for 15 min; afterwards, 50 µL of 25 mM NH_4_HCO_3_ were added to cover the gel pieces completely. Digestion was carried out overnight at 37 °C. Peptides were extracted following a short protocol: the supernatant was transferred into a new Eppendorf tube. The gel pieces were covered with an extraction solution (formic acid, 100 mM NH_4_HCO_3_ and ACN, 5:45:50, *v*/*v*/*v*) and incubated for 5 min at 37 °C with shaking. The supernatant was pooled with the overnight incubation mixture, and the gel pieces were covered with the new portion of the extraction solution. The gels were then sonicated for 10 min in an ultrasonic bath, and the obtained supernatant was pooled with the previous ones. The final solution was lyophilized to dryness and resuspended in 25 μL of a solution containing formic acid, ACN, and water (0.1:2:97.9, *v*/*v*/*v*). Peptides were analyzed using an AmaZon SL mass spectrometer (Bruker-Daltonics, Bremen, Germany) coupled with a nanoHPLC UltiMate 3000 RS (Thermo, Sunnyvale, CA, USA) and a nanoFlow-ESI nano-sprayer. The peptides were injected onto a C18 precolumn (Acclaim PepMap Nano trap Column, 3 μm, 100 A, 2 cm × 75 μm; Thermo Scientific, Sunnyvale, CA, USA) using the same mixture as that used for resuspension of the sample and further separated on a 15 cm × 75 μm RP column (Acclaim PepMap 75 μm, 100 A Nano Series TM Column, C18; Thermo Scientific, Sunnyvale, CA, USA) using a 5–50% gradient of ACN with 0.1% (*v*/*v*) formic acid for 45 min. The mass spectrometer was operated in the standard data-dependent acquisition (DDA) mode with fragmentation of two most intensive precursor ions. Ions were excluded from further fragmentation for 1 min after acquisition of two fragment spectra. The Mascot Generic format (.mgf) was generated by pre-processing the raw data with Data Analysis 4.0 software (Bruker-Daltonics, Bremen, Germany) and searched using an in-house Mascot server (v.2.3.0, Matrix Science, London, UK) against the non-redundant protein database of the National Centre for Biotechnology Information (NCBI) with taxonomic restriction “Eukaryota”. The following search parameters were applied: enzyme specificity—Trypsin; missing cleavages—1; fixed modification—Carbamidomethylation (C); variable modifications—Oxidation (M), peptide mass tolerance—±1.2 Da; fragment mass tolerance—±0.6 Da, ^13^C = 1, peptide charge: +2, +3 and +4, instrument: ESI-TRAP.

### 4.12. Other Methods

The hemolymph proteins were separated using Tris-glycine SDS-PAGE in 13.8% polyacrylamide gels according to [104]. After separation, the proteins were stained for 25 min in a 0.1% solution of Coomassie Brilliant Blue R-250 in 50% methanol and 10% acetic acid and destained in 10% acetic acid [106]. The hemolymph methanolic extracts containing proteins with molecular weight below 30 kDa and peptides were separated using Tris-tricine SDS-PAGE [107]. The gels were fixed for 30 min in a solution of 50% methanol and 10% acetic acid, and then stained for 1 h in a 0.025% solution of Coomassie Brilliant Blue G-250 in 10% acetic acid. To visualize the protein and peptide bands, the gels were rinsed in 10% acetic acid for 30 min, followed by deionized water.

The protein concentration was determined with the Bradford method [108]. The gel and membrane images were documented using the ChemiDoc™ Imaging System (Bio-Rad, Hercules, CA, USA).

### 4.13. Statistical Analysis

The statistical significance of the differences in the mean values between the groups was assessed using the single- and multifactorial analysis of variance (ANOVA) methods [109]. The statistical significance ranges of the results are marked with asterisks: * *p* < 0.05, ** *p* ≤ 0.01, *** *p* ≤ 0.001. Calculations were made using the Statistica program (TIBCO Statistica, Palo Alto, Santa Clara, CA, USA).

## Figures and Tables

**Table 1:** Identification of *G. mellonella* hemolymph proteins binding to *A. niger* α-1,3-glucan. The number of particular protein band corresponds to number presented in Figure 8.

Protein Band No.	Approximate Molecular Mass (from SDS-PAGE)	Identified N-Terminal Sequence (Bands 1–5) Identification Based on nanoLC-MS/MS (Bands 6–7)	Identification of the Protein in *Galleria mellonella*	Most Similar Protein from the Other Organism
			(Protein identity, database accession number, percent of identical amino acid residues with identified N-terminal sequence/the sequence coverage percent *)	
**1**	30 kDa	IIGDKDGEAK FGEFPWMVAI	*Galleria mellonella* phenoloxidase- activating factor 2, NCBI XP_026749280.1, 100%	*Zerene cesonia* phenoloxidase- activating factor 2-like isoform X1, NCBI XP_038221010.1, 95%
**2**	37kDa	LKDGPCPSYM DTCCLSPDRR	*Galleria mellonella* unidentified protein, whole genome shotgun sequence, GenBank NHTH01000050.1, 100%	*Papilio polytes* serine protease 44 isoform X1, NCBI XP_013146563.1, 89%
**3**	55 kDa	ETAAVFPRGI PRTPIIIPRD	*Galleria mellonella* prophenoloxidase subunit 2, GenBank AAQ75026.1, 100%	*Helicoverpa armigera* phenoloxidase subunit 2-like, NCBI XP_021182404.1, 80%
**4**	57 kDa	YEVPPAKLEA IWPKGLRVSL	*Galleria mellonella* beta-1,3-glucan recognition protein, GenBank CAK22401.1, 100%	*Papilio xuthus* beta-1,3-glucan- binding protein, GenBank KPI97191.1, 95%
**5**	61 kDa	YVVPAAKLEA IYPKGLRVSI	*Galleria mellonella* unidentified protein, whole genome shotgun sequence, GenBank NHTH01000079.1, 100%	*Plodia interpunctella* beta-1,3-glucan- binding protein, UniProtKB/Swiss-Prot Q8MU95.1, 95%
**6**	75kDa	(29–40) KLDPSLLNIQTK (41–49) VLSLLENWK (77–87) EVVTEFLSLYK (282–291) LANGLGEIPR	*Galleria mellonella* Arylphorin, Uniprot ID: Q24995_GALME, Mascot score: 170, sequence coverage 6%	
**7**	250 kDa	(40–50) ANAPQAENLKK (306–323) LGISPEFGLQITNEISEK (341–353) YHLNIYSHPELGK (406–415) FLVDVTGSDK (471–481) FVLQVSPSSFK (482–490) LLLDTPIVK (491–501) VIELEGTAVVK (612–622) QLGEEINSDFK (792–804) ETQNSVVTALQAK (854–861) FDEQAQLR (941–951) SLDPFDEIPAK (1017–1035) NNGQVAVNGASHGYPVEEK (1100–1112) ISTSESEFGNSYR (1162–1177) QACIHAVAGNAENALR (1287–1298) FPYPIVYDTDLK (1331–1340) TIANVIDEIR (1479–1491) QFLLAAANAITQR (1492–1512) ILQESIVEECVCNYANPFVGR	*Galleria mellonella* Apolipophorin, Uniprot ID: Q68YP1_GALME, Mascot score: 794, sequence coverage 15.2%	

* Percent of identical amino acid residues with identified N-terminal sequences presented only for Edman sequencing. In case of mass spectrometry data, the sequence coverage percent is shown.

## Data Availability

The data presented in this study are available on request from the corresponding author.

## Supplementary Materials

The following are available online: Figure S1. Immunodetection of α-1,3-glucan in *Aspergillus niger* cell walls; Figure S2. *Aspergillus niger* α-1,3-glucan particles imaged with AFM; Figure S3. Antibacterial activity in the hemolymph of *G. mellonella* larvae after immunization with *A. niger* α-1,3-glucan, *A. niger* conidia, or laminarin; Figure S4. Protein/peptide profiles of hemolymph methanolic extracts of *G. mellonella* larvae treated with laminarin.

## Sample Availability

Samples of the compounds are not available from the authors.
