# Supplementary material for: Fungal α-1,3-Glucan as a New Pathogen-Associated Molecular Pattern in the Insect Model Host Galleria mellonella
Source: Molecules. 2021 Aug 23;26(16):5097. doi: 10.3390/molecules26165097 (PMC8399224; doi:10.3390/molecules26165097)
Supplement: Supplementary file 1 [file molecules-26-05097-s001.zip › molecules-1318690-supplementary.pdf]

Fungal α-1,3-glucan as a new pathogen-associated molecular pattern in the insect model host *Galleria mellonella*

Sylwia Stączek [1*], Agnieszka Zdybicka-Barabas [1*], Iwona Wojda [1], Adrian Wiater [2], Paweł Mak [3], Piotr Suder [4], Krzysztof Skrzypiec [5] and Małgorzata Cytryńska [1]

[1] Department of Immunobiology, Institute of Biological Sciences, Faculty of Biology and Biotechnology, Maria Curie-Skłodowska University, Akademicka 19 St., 20-033 Lublin, Poland;
[2] Department of Industrial and Environmental Microbiology, Institute of Biological Sciences, Faculty of Biology and Biotechnology, Maria Curie-Skłodowska University, Akademicka 19 St., 20-033 Lublin, Poland;
[3] Department of Analytical Biochemistry, Faculty of Biochemistry, Biophysics and Biotechnology, Jagiellonian University, Gronostajowa 7 St., 30-387 Krakow, Poland;
[4] Department of Analytical Chemistry and Biochemistry, Faculty of Materials Sciences and Ceramics, AGH University of Science and Technology, Mickiewicza 30 Ave., 30-059 Krakow, Poland;
[5] Analytical Laboratory, Faculty of Chemistry, Maria Curie-Skłodowska University, M.C. Skłodowska Square 5, 20-031 Lublin, Poland;

* Correspondence: s.staczek@poczta.umcs.lublin.pl (S.S.); barabas@poczta.umcs.lublin.pl (A.Z.-B.)

Content:

Figure S1. Immunodetection of α-1,3-glucan in *Aspergillus niger* cell walls.

Figure S2. *Aspergillus niger* α-1,3-glucan particles imaged with AFM.

Figure S3. Antibacterial activity in the hemolymph of *G. mellonella* larvae after immunization with *A. niger* α-1,3-glucan, *A. niger* conidia, or laminarin.

Figure S4. Protein/peptide profiles of hemolymph methanolic extracts of *G. mellonella* larvae treated with laminarin.

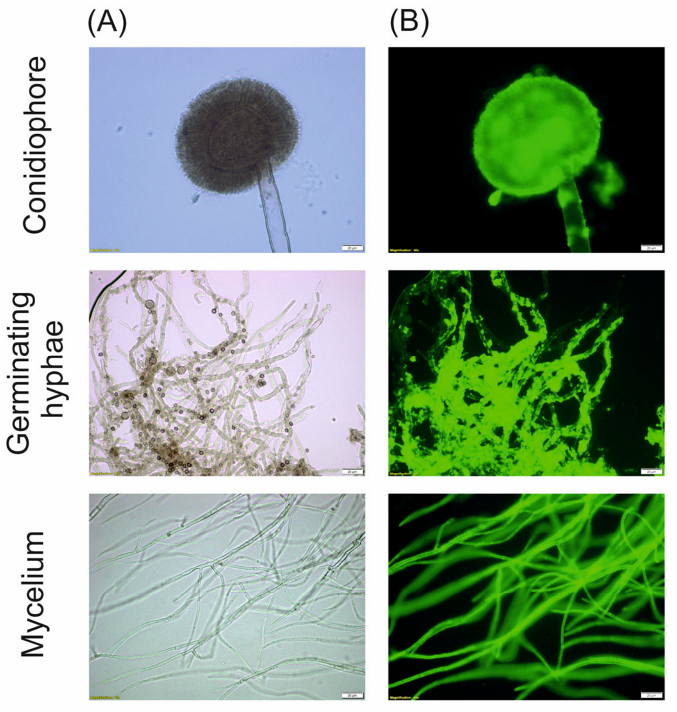

Figure S1. Immunodetection of α-1,3-glucan in *Aspergillus niger* cell walls. The mycelium and conidiophores were fixed on microscopic slides and incubated with mouse antibodies against α-1,3-glucan and then with goat anti-mouse Alexa-Fluor 488 labeled antibodies. Next, they were imaged using a laser scanning confocal microscope. Column A - images in transmitted light, column B - images showing the presence of α-1,3-glucan (green fluorescence). Scale - 20 μm.

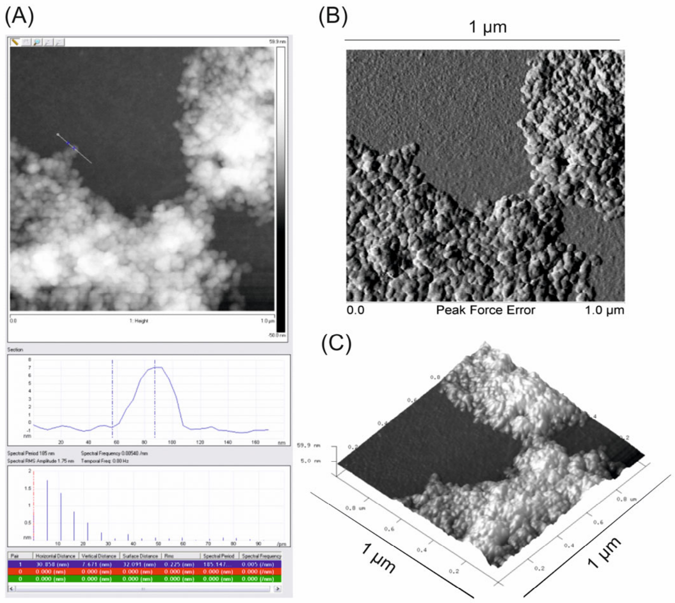

Figure S2. *Aspergillus niger* α-1,3-glucan particles imaged with AFM. The height image (A), the surface topography (B), and the three-dimensional image (C) of α-1,3-glucan particles in the suspension are shown. The bottom of panel (A) shows the surface profile corresponding to the white line in the height image.

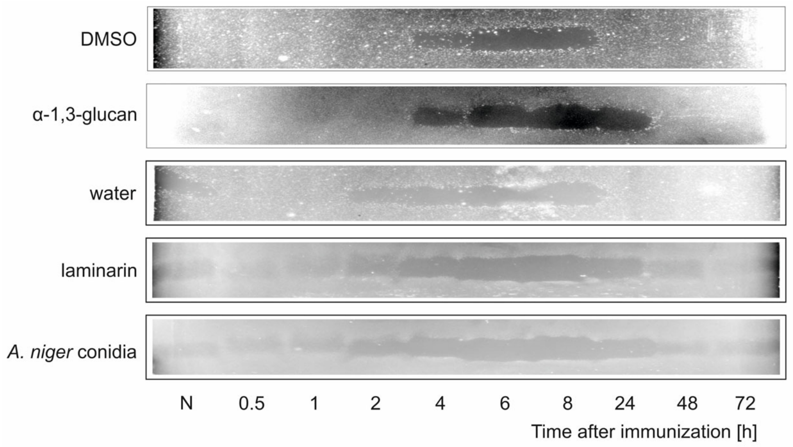

Figure S3. Antibacterial activity in the hemolymph of *G. mellonella* larvae after immunization with *A. niger* α-1,3-glucan, *A. niger* conidia, or laminarin. The larvae were immunized with α-1,3-glucan (5 μg), conidia (1×10^5), or laminarin (50 μg). The control larvae were injected with DMSO or water as described in Materials and methods. Hemolymph was collected at the indicated time points and analyzed using bioautography. Fragments of gels presenting zones of *E. coli* growth inhibition (shaded areas) are demonstrated. N – non-immunized larvae.

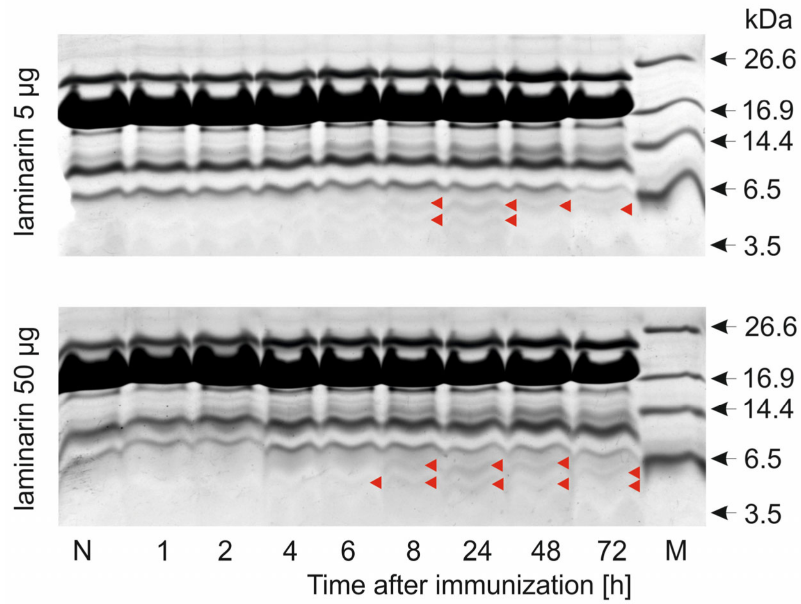

Figure S4. Protein/peptide profiles of hemolymph methanolic extracts of *G. mellonella* larvae treated with laminarin. The larvae were injected with two doses of laminarin (5 μg and 50 μg). The hemolymph was collected at the indicated time points; the methanolic extracts were obtained and separated by Tris-tricine SDS-PAGE as described in Materials and methods. N – non-immunized larvae; M - molecular mass markers. Peptide bands appearing in the hemolymph after immunization are indicated with red arrowheads.
